# Molecular Imaging of Stems Cells: In Vivo Tracking and Clinical Translation 

**DOI:** 10.1155/2017/1783841

**Published:** 2017-01-02

**Authors:** Stefania Rizzo, Francesco Petrella, Letterio S. Politi, Ping Wang

**Affiliations:** ^1^Department of Radiology, European Institute of Oncology, Milan, Italy; ^2^Department of Thoracic Surgery, European Institute of Oncology, Milan, Italy; ^3^Department of Hematology/Oncology and Department of Radiology, Boston Children's Hospital, Boston, MA, USA; ^4^Department of Radiology, University of Massachusetts Medical School and University of Massachusetts Memorial Medical Center, Worcester, MA, USA; ^5^Molecular Imaging Laboratory, MGH/MIT/HMS Athinoula A. Martinos Center for Biomedical Imaging, Department of Radiology, Massachusetts General Hospital, Harvard Medical School, Boston, MA, USA

Once upon a time, there were diseases for which patients had to die without cure and to be treated only to relieve or retard symptoms, such as diabetes, myocardial infarction, postsurgical bronchopleural fistulas, Parkinson's disease, and Alzheimer's disease.

Then stem cells started to be intensively studied for infusion or transplantation into tissues for purposes of repair, revascularization, and other therapeutic actions [1, 2]. After systemic or local administration, stem cells may proliferate, migrate, and repopulate pathologic sites, bringing remarkable therapeutic effect. Indeed, several studies have demonstrated the capacity of adult stem cell transplantation to restore/induce bone repair and revascularization of the ischemic cardiac tissue in vivo, while investigations are underway on tissue neuroregeneration in disorders such as Parkinson's and Alzheimer's disease and diseases of the lung and airways [3–5], liver [6], diabetes, and other organs.

A risk that has been identified in early stem cell studies lays in the ability of undifferentiated human embryonic stem cells to produce tumors in vivo, such as teratomas and teratocarcinomas [7]. As a result, stem cell misbehavior after delivery has been regarded as a major obstacle for translation of stem cell-based therapies into clinical reality.

Furthermore, it has been demonstrated that, when injected systemically, mesenchymal stromal cells (MSCs) accumulate in the lungs and capillary beds of other tissues, thus decreasing the number of MSCs migrating to target areas for treatment [8]. Molecular imaging can offer a better understanding of cell fate after transplantation, thus providing successful implementation of cell therapies.

For instance, J. Cao et al. demonstrated allogenic bone marrow MSCs home to the dorsal skin, apart from the lungs and kidneys, after tail-vein-injection, could not be detected 14 days later. M. Song et al. were able to track systemically transplanted human bone marrow-derived mesenchymal stromal cells mice with smoke inhalation injury through BLI, eventually demonstrating that MSC xenografts repaired smoke inhalation-induced lung injury in mice.

Magnetic Resonance Imaging (MRI), Positron Emission Tomography (PET), Single-Photon Emission Computed Tomography (SPECT), Fluorescence Imaging (FLI), and Bioluminescence Imaging (BLI) are multiple examples of imaging systems that can visualize signals generated form labelled cells, thus providing accurate and detailed information about cell fate, migration, and engraftment following transplantation.

In the specific setting of MRI, X.-G. Peng et al. demonstrated that Diffusion Tensor Imaging could be a useful tool for noninvasive evaluation of muscle tissue damage and repair in animal models and patient with ischemic diseases, while X. Chen et al. demonstrated that iron particles are not a reliable marker for in vivo tracking the fate of MSCs engraftment in case of myocardial infarction.

Cell tracking can be performed either by molecular probes entering the target cell by active/passive transport or by overexpression of reporter genes integrating into cellular genome [9].

As demonstrated by R. Donders et al., two-photon confocal laser scanning microscopy (TPM) and second harmonic generation (SHG) are alternative techniques that may enable the detection of cells and extracellular structures, based on intrinsic properties of the specific tissue and intracellular molecules under optical irradiation.

Molecular imaging may also play a role in defining the proper cell type, delivery method, cell dose, therapeutic window, and evaluation of toxicity to patients, by identification of early transformation of cell grafts into tumors, as well as imaging the proliferation and/or expression of tumor-specific markers, which cannot be detected by traditional imaging techniques.

Moreover, in vivo imaging of stem cells may disclose how cells survive and proliferate within the target tissue, as well as their differentiation and maturation, thus providing precious data to generate a dose-response curve to identify the optimal dose and dosing frequency of cell therapies [9].

For instance, in the specific setting of stem cell transplantation for liver diseases, there have been two main clinical applications of molecular imaging [10, 11]. In this special issue of this journal, the multiple possibilities of monitoring stem cell transplantation for liver diseases have been extensively exposed in a review article by P. Wang et al.

However, the serial visualization and tracking of transplanted stem cells, including their possible migration and/or retention in other sites, are still issues to be resolved before preclinical studies can be turned into clinical studies. For example, ultrasound-guided intralesional injection of MSCs is held as the benchmark for cell delivery in tendonitis because many reports have determined that local injury retains cells within a small radius of the site of injection. However, in this issue, A. Scharf et al. have demonstrated that there is a greater delocalization than expected, and relatively few cells are retained within collagenous tendon compared to surrounding fascia, underlying the need of further in vivo studies.

Similar issues are still unsolved about the use of extracellular vesicles (EVs), considered as paracrine mediators of the beneficial effects on tissue remodeling associated with cell therapy. The administration of MSCs-derived EVs may have the potential to open new and safer therapeutic avenues, alternative to cell-based approaches, for degenerative diseases, but studies about the biodistribution upon systemic delivery of EVs indicate in liver, spleen, and lungs preferential target organs. With this regard, G. Di Rocco et al. reviewed the existing strategies for in vivo tracking and targeting of EVs isolated from different cellular sources and the studies elucidating the biodistribution of exogenously administered EVs.

Although many examples of in vitro and in vivo studies have already been published, clinical applications of molecular imaging in stem cells therapies are still limited.

At the moment we are writing in the US more than 3,900 clinical trials with “stem cell transplantation” registered (https://www.clinicaltrials.gov), 1,384 of which are open and are recruiting. Therefore, it is our opinion that in the forthcoming years the application of cell tracking studies in clinical research will dramatically increase and that the information gathered through cellular and molecular imaging techniques will play an important role in clinical trials design, in monitoring the cell delivery, in defining the fate of the transplantation, in interpreting the clinical data, and in understanding the reasons of success or failure of the trials.

After further evaluation of different possibilities of tracking stem cells, we do expect that many clinical questions, raised from applications of stem cells-based therapies, will find an answer in molecular imaging. Therefore, we do believe that stem cell-based therapies and molecular imaging will live together happily ever after.

*Stefania Rizzo* *Francesco Petrella* *Letterio S. Politi* *Ping Wang*
